# Carbon Stocks of Tropical Coastal Wetlands within the Karstic Landscape of the Mexican Caribbean

**DOI:** 10.1371/journal.pone.0056569

**Published:** 2013-02-14

**Authors:** Maria Fernanda Adame, J. Boone Kauffman, Israel Medina, Julieta N. Gamboa, Olmo Torres, Juan P. Caamal, Miriam Reza, Jorge A. Herrera-Silveira

**Affiliations:** 1 Centro de Investigación y Estudios Avanzados (CINVESTAV) del Instituto Politécnico Nacional (CINVESTAV-IPN), Mérida, México; 2 Australian Rivers Institute, Nathan Campus, Griffith University Brisbane, Queensland, Australia; 3 Department of Fisheries and Wildlife, Oregon State University, Corvallis, Oregon, United States of America; 4 The Center for International Forest Research, Bogor, Indonesia; 5 Colectividad Razonatura A.C., Mexico City, México; 6 Amigos de Sian K'aan, Cancún, México; Lakehead University, Canada

## Abstract

Coastal wetlands can have exceptionally large carbon (C) stocks and their protection and restoration would constitute an effective mitigation strategy to climate change. Inclusion of coastal ecosystems in mitigation strategies requires quantification of carbon stocks in order to calculate emissions or sequestration through time. In this study, we quantified the ecosystem C stocks of coastal wetlands of the Sian Ka'an Biosphere Reserve (SKBR) in the Yucatan Peninsula, Mexico. We stratified the SKBR into different vegetation types (tall, medium and dwarf mangroves, and marshes), and examined relationships of environmental variables with C stocks. At nine sites within SKBR, we quantified ecosystem C stocks through measurement of above and belowground biomass, downed wood, and soil C. Additionally, we measured nitrogen (N) and phosphorus (P) from the soil and interstitial salinity. Tall mangroves had the highest C stocks (987±338 Mg ha^−1^) followed by medium mangroves (623±41 Mg ha^−1^), dwarf mangroves (381±52 Mg ha^−1^) and marshes (177±73 Mg ha^−1^). At all sites, soil C comprised the majority of the ecosystem C stocks (78–99%). Highest C stocks were measured in soils that were relatively low in salinity, high in P and low in N∶P, suggesting that P limits C sequestration and accumulation potential. In this karstic area, coastal wetlands, especially mangroves, are important C stocks. At the landscape scale, the coastal wetlands of Sian Ka'an covering ≈172,176 ha may store 43.2 to 58.0 million Mg of C.

## Introduction

Tropical wetlands are one of the most carbon (C) rich ecosystems in the world. The organic-rich soils of many mangroves and tidal marshes contain exceptionally large C stocks [1], [2] that can be two to three times higher than those measured in most terrestrial forests. For example, the IPCC [3] default values for tropical and temperate forests are <400 Mg ha^−1^, whereas mangrove mean carbon stocks can exceed 1,100 Mg ha^−1^ [1]. Conservation and restoration of coastal wetlands are a priority for maintaining C stocks and preventing emissions arising from wetland loss [4], [5].

Mangroves have among the highest rates of deforestation of any forest ecosystem [6]. Land conversion has resulted in the loss of over one third of all mangroves over the past 20–50 years [7], [8]. Dominant causes of deforestation and degradation include: agriculture and aquaculture conversion, pollution, coastal development, and hydrological disruptions [7], [9]. Besides the loss of aboveground biomass following mangrove disturbance, decomposition of organic material causes the release of considerable amounts of CO_2_ to the atmosphere [10]. Given the large C stocks of mangroves, the emissions arising from conversion are likely exceptionally high and a significant source of greenhouse gasses [1]. Furthermore, global climate change may affect mangrove cover and distribution through an increase in sea-level rise, changes in tropical storm intensity, and changes in stream and groundwater flows that discharge into mangroves [11]. Because of their large ecosystem C stocks, their vulnerabilities to land use, and the numerous other ecosystem services they provide, coastal wetlands are of increasing interest for participation in climate change mitigation strategies [12]. To participate in climate change mitigation strategies, such as Reduced Emissions from Deforestation and Degradation (REDD+ [13]), it is necessary to determine C stocks and emissions baselines.

Along the eastern coast of the Yucatan Peninsula, wetlands are composed of a mosaic of mangroves and herbaceous-dominated marshes. Mangroves are largely dominated by *Rhizophora mangle* and occur as different structural forms, from tall forest to dense shrub lands. The distinct communities of coastal wetlands that characterize the eastern Yucatan Peninsula are reflective of specific geological characteristics of the region. The Yucatan Peninsula is an oligotrophic karstic setting [14] with a highly permeable carbonate substrate and a complex subsurface hydrologic system that transports freshwater to coastal wetlands where it mixes with seawater [15]. As a result of the carbonate rich substrate of the region, groundwater is low in phosphorus (P), thus primary productivity of coastal wetlands in the area is greatly influenced by P availability [16], [17].

In this study, we measured whole-ecosystem C stocks of different coastal wetlands within the Sian Ka'an Biosphere Reserve (SKBR) in the Yucatan Peninsula. In this relatively pristine location, we measured C stocks of tall, medium, and dwarf mangroves, as well as coastal marshes. Our objectives were to determine and compare ecosystem C stocks of different vegetation types, and to determine abiotic factors that could affect their C storage potential. We hypothesized that: 1) Coastal wetlands in SKBR are a significant C stock, 2) Highest C stocks are found in tall mangroves, 3) Most of the C within the wetlands is stored in the soil, and 4) Soil P is closely associated to C stock size. This study provides the first whole-ecosystem C stock analysis of different types of coastal wetlands within a tropical karstic zone. Mangroves in karstic regions could account for more than 1.5 million ha (>10% of total mangrove cover), notably, in Cuba (>400,00 ha), the Yucatan Peninsula, Mexico (>300,000 ha), Madagascar (>250,000 ha), and the Philippines (>250,000 ha) [8], [18]–[21]. The C stocks calculated in this study are invaluable information for C stock baselines of coastal wetlands of the Yucatan Peninsula and similar coastal settings.

## Methodology

### 1. Study site

The SKBR is located in Quintana Roo State in the Yucatan Peninsula, Mexico. The SKBR is both a UNESCO World Heritage site established in 1986 and a Ramsar site [22]. The Reserve covers an area of 551,715 ha that includes evergreen and deciduous upland forests, savannahs, and a large expanse of coastal wetlands (>170,000 ha, [23], [24]). The coastal wetlands of the area are flooded by a mixture of seawater from tidal fluxes and fresh groundwater from subsurface flows through the karstified limestone [25]. Coastal wetland plant communities in the SKBR were separated following classifications of Lugo and Snedaker [26] and Murray et al. [27] into the following: a) tall mangroves with a mean height >5 m, which can be associated with fresh water springs; b) medium mangroves that form dense stands of trees of 3 to 5 m in height, usually as fringing forest and c) dwarf mangroves, composed of dense stands of trees whose height is <1.5 m. Additionally, herbaceous dominated marshes of *Typha domingensis*, *Cladium jamaicense*, *Eleocharis cellulosa*, and *Eleocharis interstincta* cover extensive coastal areas [28].

The climate of the SKBR is warm, sub humid with most precipitation occurring in the summer months. The mean annual temperature of the region is 26°C, with a mean annual minimum and maximum of 20 and 31°C, respectively (Tulum Meteorological Station, 1971–2000 [29]). Mean annual precipitation is 1588 mm, 1971–2000 [29]). The SKBR receives frequent tropical storms and hurricanes (14 tropical storms and 6 hurricanes from 1857 to 2009 [30]).

### 2. Field sampling

**Figure 1:**
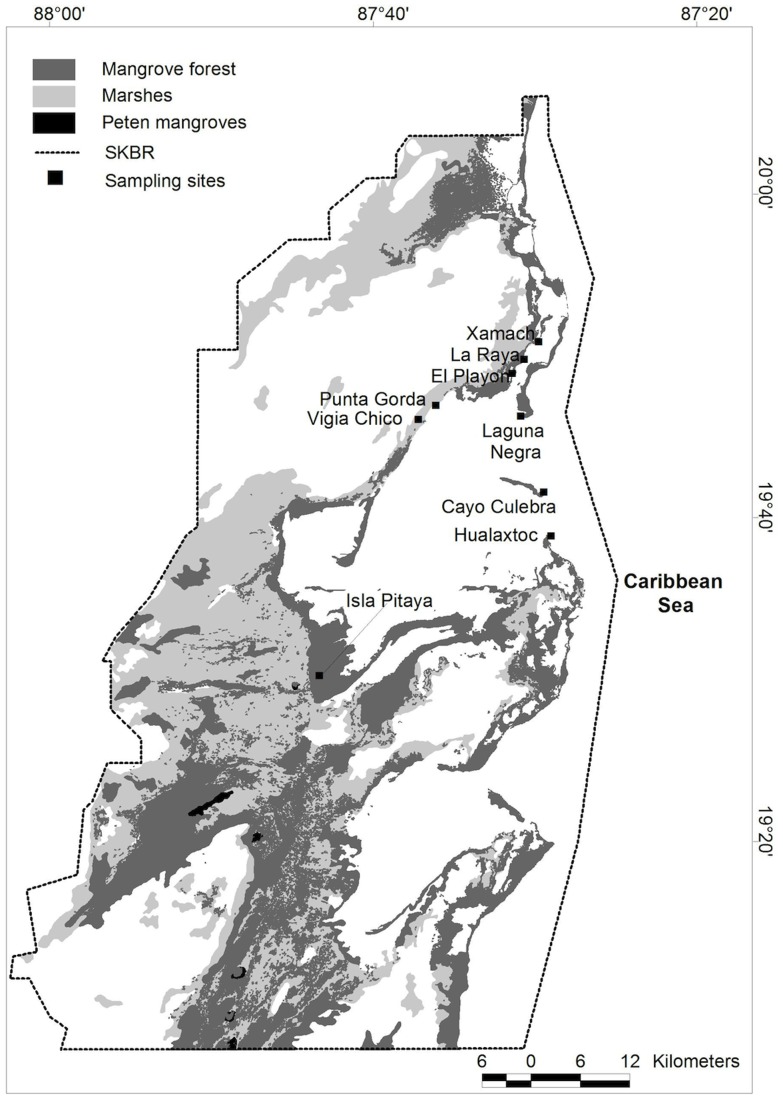
Sample locations within Sian Ka'an Biosphere Reserve. Mangrove forest area map (dwarf+medium+tall) was obtained from CONABIO [23]; the map for “Peten” mangroves (tall mangroves associated with freshwater springs) was obtained from the Series III, INEGI (2005) and the map of marshes from INEGI (2000) [24].

During August 2011, we sampled 9 different coastal wetland sites within the SKBR that represented four kinds of vegetation types: a) tall mangroves (2 sites); b) medium mangroves (2 sites); c) dwarf mangroves (3 sites); and d) marsh (2 sites) (Fig. 1,Table 1). Within each site, we measured whole-ecosystem C stocks following methodologies outlined by Kauffman and Donato [31]. At each sampled site, six plots were established 25 m apart along a 125 m transect established in a perpendicular direction from the marine ecotone. At each plot, we collected data necessary to calculate total C stocks derived from standing tree biomass, downed wood (dead wood on forest floor) and soil. We also sampled soils for N and P concentration and interstitial salinity.

**Table 1:** Characteristics of sampling locations.

Site	Latitude/Longitude	Canopy height (m)	Mean diameter (cm)	Density (tree ha^−1^)	Salinity	Dominant species
Tall mangroves						
Isla Pitaya	19.4867					*L. racemosa* (64%)
	-87.7004	3–10	9.8 (0.6)	3,183 (336)	28.6 (7.0)	*R. mangle* (28%)
Cayo Culebra	19.6957 -					
	-87.4659	3–14	7.8 (0.5)	6,843 (2,460)	38.9 (0.5)	*R. mangle* (96%)
Medium mangroves						
Hualaxtoc	19.6477					
	-87.4540	2–11	4.1 (0.6)	9,409 (3,023)	n.a.	*R. mangle* (84%)
Laguna Negra	19.7800					
	-87.4789	2–5	3.9 (0.3)	11,406 (2,191)	44.9 (1.0)	*R. mangle* (94%)
Dwarf mangroves						
Xamach	19.8612					*R. mangle* (96%)
	-87.4612	0.4–1.5	2.1 (0.1)	8,886 (1,430)	57.2 (5.5)	*A. germinans* (33%)
La Raya	19.8408					
	-87.4800	0.1–1.3	1.4 (0.0)	37,932 (12,595)	n.a.	*R. mangle* (100%)
El Playon	19.8218					
	-87.4950	0.6–1.4	1.1 (0.1)	47,216 (11,922)	49.6 (1.6)	*R. mangle* (100%)
Marsh						
Punta Gorda	19.7936					*T. domingensis*
	-87.5743	1–2	n.a.	n.a.	5.2 (0.8)	*R. mangle*
Vigia Chico	19.7757					*Cladium jamaicense*
	-87.5887	1–2	3.2 (0.2)	3,183 (336)	8.5 (1.6)	*C. erectus*

Nomenclature of vegetation type follows the classification by Murray et al. [27]. Values are shown as mean (standard error); n.a. = not available.

#### 2.1. Biomass of trees and shrubs

Composition, tree density, and basal area in tall and medium mangroves were quantified through measurements of the species and diameter at 1.3 m height (DBH) of all trees rooted within each plot of each transect. Plot size for tree measurements in the tall and medium mangroves was 154 m^2^ (radius of 7 m), except in the Laguna Negra, a site of medium sized mangroves where tree density exceeded 8,000 trees ha^−1^. In this dense forest, a 2 m radius plot was sufficient to accurately quantify the small diameter tree biomass [31]. Similarly, due to the lower density of the dwarf mangroves of Xamach, tree density was measured in six 2 m radius plots. In the dwarf mangroves sites of El Playon and La Raya tree density exceeded 30,000 trees ha^−1^. In these sites, tree mass was determined using six semicircular 2 m radius plots (one semicircle to the right of the transect alternated with one to the left) following methods outlined in by Kauffman and Donato [31]. The diameter of trees of *R. mangle* was measured at the main branch, above the highest prop root (D_R_). In dwarf mangroves, the diameter of the main branch of the tree was measured at 30 cm from the ground (D_30_). Additionally, in the dwarf mangroves we measured tree height, and length and width of the crown, following guidelines by Ross et al. [32]. Grass and sedge biomass in the marsh communities was determined through harvest of all aboveground materials within two 20×20 cm quadrants within each of the 6 plots (n = 12 quadrats). The wet mass was determined in the field and then a subsample was collected from each quadrant and oven-dried to determine dry weight of marsh vegetation.

Allometric equations were used to calculate tree biomass for each site (Table 2). In the tall mangroves, we used formulas provided in Smith and Whelan [33]. For dwarf mangroves, we compared the formula of Ross et al. [32] that used crown volume with the formula of Cintron and Shaeffer-Novelli [34] that used main stem diameter and tree height (Biomass (g) = 125.9571 D_30_^2^ * Height (m)^0.8557^). The biomass estimations calculated with the formula of Cintrón and Shaeffer Novelli were 12.3% higher (*F*_1, 773_ = 5.41, *p*≤0.0001). Following the IPCC Good Practice Guidelines, we report biomass of dwarf mangroves using the conservative estimations obtained with the formula of Ross et al. Belowground root biomass for mangrove trees was calculated using the formula by Komiyama et al. [35] using the wood density values from Zanne et al. [36] (Table 2). Tree C was calculated from biomass by multiplying by a factor of 0.48 for aboveground and 0.39 for belowground biomass, as recommended by Kauffman and Donato [31]. The C content of the aboveground mass of marshes was calculated using a factor of 0.45 of the total [31].

**Table 2:** Allometric equations used to calculate aboveground and belowground biomass of mangrove trees.

Aboveground biomass		
Tall and medium mangroves		Reference
*R. mangle*	Biomass: log_10_ B = 1.731*log_10_ D_R_ - 0.112	Smith and Whelan [33]
	Leaves: log_10_ B = 1.337*log_10_ D_R_ - 0.843	
	Stem: log_10_ B = 1.884*log_10_ D_R_ - 0.510	
	Branch: log_10_ B = 1.784*log_10_ D_R_ - 0.853	
	Prop roots: log_10_ B = 0.160 *log_10_ D_R_ - 1.041	
*A. germinans*	Biomass: log_10_ B (kg) = 1.934*log_10_ DBH (cm) - 0.395	
	Leaves: log_10_ B = 0.985*log_10_ DBH - 0.855	
	Stem: log_10_ B = 2.062*log_10_ DBH - 0.590	
	Branch: log_10_ B = 1.607*log_10_ DBH - 1.090	
*L. racemosa*	Biomass: log_10_ B (kg) = 1.930*log_10_ DBH (cm) - 0.441	
	Leaves: log_10_ B = 1.160*log_10_ DBH - 1.043	
	Stem: log_10_ B = 2.087*log_10_ DBH - 0.692	
	Branch: log_10_ B = 1.837*log_10_ DBH - 1.282	
Dwarf mangroves		
*R. mangle*	Ln B (g) = 2.528+(1.129 (Ln D_30_^2^ (cm))+(0.156* Ln Crown Volume (cm^3^))	Ross et al. [32]
*A. germinans*	Ln B (g) = 2.134+(0.895 (Ln D_30_^2^ (cm))+(0.184* Ln Crown Volume (cm^3^))	
Belowground biomass		
All mangroves		
*R. mangle*	B (kg) = 0.196*(1.05^0.899^)* (D_R_^2^)^1.11^	Komiyama et al. [35]
*A. germinans*	B (kg) = 0.196*(0.90^0.899^)* (DBH^2^)^1.11^	
*L. racemosa*	B (kg) = 0.196*(1.05^0.899^)* (DBH^2^)^1.11^	

B = biomass; D_R_ = diameter above highest prop root; DBH = diameter at breast height; D_30_ = diameter at 30 cm from the ground. Wood density values used for calculating belowground biomass were obtained from Zanne et al [36].

Standing dead trees were included in our calculations. For each dead tree, the stem diameter was measured and assigned to one of three decay class described in Kauffman and Donato [31]: 1- dead trees without leaves, 2- dead trees without secondary branches, and 3- dead trees without primary or secondary branches. The biomass for each tree status was calculated using allometric equations of plant components. For dead trees of Status 1, biomass was calculated as the total dry biomass minus the biomass of leaves. The biomass of trees of Status 2 was calculated for *R. mangle* as the sum of stem, branches and prop roots and for *Laguncularia racemosa* and *Avicennia germinans* as the sum of stem and branches. Finally, the biomass of trees of Status 3 was calculated as the biomass of the main stem (Table 2). Standing dead trees in the dwarf forests were very rare and were included with live trees if present.

#### 2.2. Downed wood

We used the planar intersect technique [37] adapted for mangroves [31] to calculate mass of dead and downed wood. At the center of each plot, four 14 m transects were established. The first was established in a direction that was 45° off the direction of the main transect. The other three were then established in directions that were 90° off from the previous transect. At each transect, the diameter of any downed, dead woody material (fallen/detached twigs, branches, prop roots or stems of trees and shrubs) intersecting the transect was measured. Wood debris >2.5 cm but <7.5 cm in diameter (hereafter “small” debris) at the point of intersection was measured along the last 5 m of the transect. Wood debris >7.5 cm in diameter (hereafter “large” debris) at the point of intersection was counted from the second meter to the end of the transect (12 m in total). Large downed wood was separated in two categories: sound and rotten. Wood debris was considered rotten if it visually appeared decomposed and broke apart when kicked. To determine specific gravity of downed wood we collected ≈60 pieces of down wood of different sizes (small, large-sound, and large-rotten) and calculated their specific gravity as the oven-dried weight divided by its volume. Using the specific gravity for each group of wood debris, biomass was calculated using formulas reported in Kauffman et al. [38]. Downed wood was converted to C using a factor of 0.50 as recommended by Kauffman et al. [39].

#### 2.3. Soil carbon and nutrients

At each plot, soil samples for bulk density and nutrient concentration were collected using a peat auger consisting of a semi-cylindrical chamber of 6.4 cm-radius attached to a cross handle. This auger is efficient for sampling undisturbed cores from soft and wet soils [1], [38]. The core was systematically divided into depth intervals of 0–15 cm, 15–30 cm, 30–50 cm, 50–100 cm and >100 cm (if parent materials were not encountered before 100 cm depth). From each core, the depth of the organic horizon, if present, and the depth to parent materials was measured. Samples of a known volume were collected in the field and then dried to constant mass to determine bulk density. Samples were sieved and homogenized. Total inorganic phosphorus (P) was determined as orthophosphates following the methods described by Aspila et al. [40] and Parsons et al. [41]. Briefly, dry 0.2 g of soil were combusted at 550°C for 2 h, followed by an extraction with 1 N HCl for 16 hours at 150 rpm. After extraction, the samples were filtered and read at 885 nm using the colorimetric method from the reaction of ortophosphates with ammonium-molybdate The concentration of C and N were determined using the dry combustion method (induction furnace) with a Leco CNS-2000 Macro Analyzer (Oregon State University Central Analytical Laboratory). Because of the karstic substrate, a significant proportion of soil C was carbonates. Carbonates can be removed before analysis by adding acid (usually HCl) to the sample. However, in some samples, the amount of carbonates was >80% of the total C, and samples required very high quantities of acid (>1 mL of HCl per 1 g of sample), resulting in inaccuracies of weight measurements due to hydration of the sample and reactions of acid with soil compounds which could lead to over or underestimations of C content [42]. Alternatively, we used a combination of techniques to differentiate between organic matter and carbonates [42]; dry combustion and the loss on ignition method (LOI). Two grams of dry soil (oven dried for 1 h at 70°C) were left in the furnace at 550°C for 4 h, re-weighted and then left for 2 more hours at 950°C [43], [44]. We calculated the percentage of organic matter from the difference between the dry weight and the weight after 550°C, and the percentage of carbonates as the difference between the weight after 550°C and the weight after 950°C. Organic C (OC) and inorganic C (IC) were calculated using conversion factors suggested by Dean [43]. Because the LOI is considered a qualitative, not a quantitative method [45], that usually overestimates C content, values obtained from LOI were corrected [46]. We multiplied the proportion of OC and IC obtained through LOI by the C content obtained from the dry combustion, from which we obtained a good approximation of organic (OC%) and inorganic carbon (IC%) for each sample.

#### 2.4. Interstitial salinity

Within each plot, we measured interstitial salinity by extracting water from the ground at 30 cm using a syringe and an acrylic tube. The syringe was rinsed twice before obtaining a clear water sample from which salinity was measured using an YSI-30 multiprobe sensor (YSI, Xylem Inc. Ohio, USA).

### 3. Scaling up

The area of mangroves (tall+medium+dwarf) was obtained from The National Commission of Biodiversity [23]. We were able to distinguish areas of some tall mangroves from medium and dwarf forests using the map of “Peten” vegetation ([24], INEGI, Series III, 2005). In the Yucatan Peninsula, Peten vegetation refers to vegetation associated with freshwater springs, which is composed of tall mangroves in coastal zones. Finally, marsh areas (referred in the data set as “Popal-Tular” vegetation) were determined from the National Forest Inventory [24]. The three vegetation layers were added into one map, being careful not to duplicate the mangrove area of tall mangroves identified as Peten vegetation. With the new map, we calculated the area of mangroves and marshes. With the available information, we were able to distinguish tall mangrove areas associated with water springs (Fig. 1), from other mangrove types. However, we could not distinguish among those dwarf, medium and tall mangroves that were not associated with freshwater springs. The mangrove area of tall mangroves associated to freshwater springs was very low (<1% of the total). The C stock estimates for the SKBR are given as a range between mangrove forests consisting of 100% dwarf dominance to 100% medium-tall dominance. Most mangroves in SKBR are dwarf forest, so it is likely that the C stock of SKBR is closer to the lower range of our calculations than to the higher range.

### 4. Statistical analyses

Differences among biomass and carbon stocks among vegetation types (tall, medium and dwarf mangroves, and marsh) were tested with Analysis of Variance (ANOVA), where vegetation type was the fixed effect, and site (nested in vegetation type) and plot (nested in site) were the random effects of the model. Differences in soil C, N, and P concentrations by depth were also tested with ANOVA, with depth as the fixed effect and site as the random effect of the model. Normality was assessed using probability plots, histograms and Shapiro-Wilk tests. When required, some variables (soil P and C stocks) were log transformed to comply with normality and homogeneity of variances when testing linear models. When transformations were not enough to achieve normality, differences among categories were analyzed using Wilcoxon Signed Rank test. When significant differences were found, pair-wise comparisons were explored using Scheffé post-hoc tests. Step-wise Multiple Regressions were used to test the effect nutrients and interstitial salinity on C stocks. Multicolinearity was assessed using a variance inflation factor (VIF), which was calculated for each parameter. Models with low VIF (<4 [47]) were selected. Analyses were performed using Data Desk (version 6.2, OSX, Ithaca, NY, USA) and SPSS Statistics (version 20, IBM, New York, USA). Throughout the manuscript, data are reported as mean ± standard error.

## Results

### 1. Vegetation types

The coastal wetlands of SKBR were composed of at least four distinct vegetation types. The first group was characterized by tall mangroves of up to 14 m in height. These mangroves formed low tree density stands (<7,000 trees ha^−1^) of *R. mangle* and occasionally *L. racemosa*, where interstitial salinity was low (<32‰). The second group corresponds to medium height mangroves, which formed dense stands (∼9,000–11,000 trees ha^−1^) of *R. mangle* trees with heights of up to 11 m, but mostly around 5 m. The third group was characterized by dwarf mangroves, which rarely reached heights >1.5 m and were composed of very dense stands (up to ∼47,000 trees ha^−1^) usually of *R. mangle*, but also of *A. germinans* in sites where interstitial salinity was high (>55‰). Finally, marshes were composed of at least two species: *T. domingensis* and *C. jamaicense*, the first one associated with sparse trees of *R. mangle* and the second with *C. erectus* (Table 1).

### 2. Tree, shrub, and graminoid biomass

**Figure 2:**
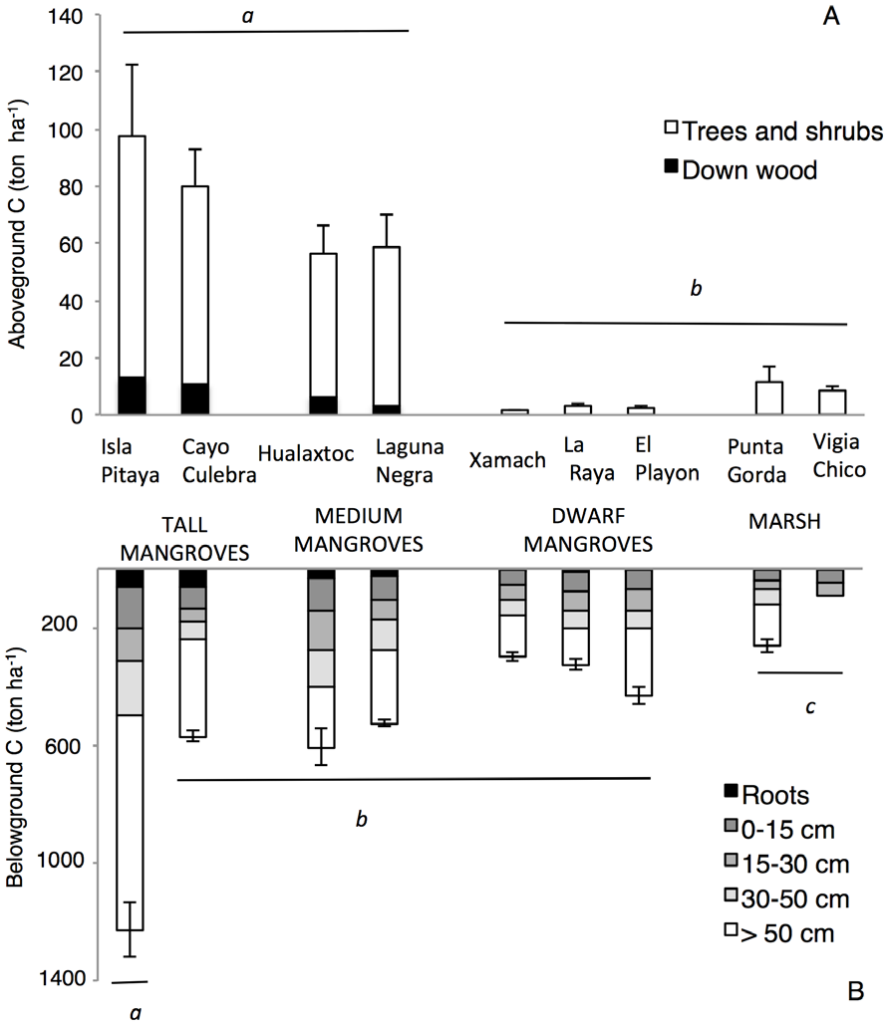
Ecosystem C stocks of coastal wetlands of Sian Ka'an Biosphere Reserve. The stocks are partitioned by A) aboveground (trees and down wood) and B) belowground (roots and soil) components. Lower case letters represent significant differences among sites and vegetation types (*n* = 6 per site, *p*≤0.0001). Note different scales between panel A and B.

Aboveground tree biomass of coastal wetlands varied by over 60-fold, ranging from 3.0±0.4 Mg ha^−1^ in a dwarf mangrove forest (Xamach) to 176.2±47.4 Mg ha^−1^ in a tall mangrove forest (Isla Pitaya) (Table 3). Biomass and aboveground C stocks in tall and medium mangroves were significantly greater than dwarf mangroves and marshes (*F*_3, 53_ = 63.52, *p* = 0.0002) (Figure 2A). The aboveground biomass of marshes often exceeded that of dwarf mangroves with a mean of 18.0±2.2 Mg ha^−1^ in Vigia Chico and 23.4±3.0 Mg ha^−1^ in Punta Gorda. The contribution of dead trees to aboveground biomass of tall mangroves was <6% in all sites except in Cayo Culebra, where the contribution was 27.3%. Belowground tree biomass was lowest in dwarf mangroves (8.7±0.9 Mg ha^−1^, Xamach) and highest in tall mangroves (156.6±44.2 Mg ha^−1^, Isla Pitaya). The overall mean C stocks of mangrove trees and shrubs (all mangrove sites combined ) was 31.9±10.9 Mg ha^−1^.

**Table 3:** Aboveground biomass, belowground biomass and total C stocks in vegetation (Mg ha^−1^).

	Biomass (Mg ha^−1^)		C (Mg ha^−1^)
Site	Aboveground	Belowground	
Tall mangroves			
Isla Pitaya	176.2 (47.4)	156.6 (44.2)	145.6 (40.0)
Cayo Culebra	144.9 (23.5)	147.2 (25.3)	127.0 (20.9)
Medium mangroves			
Hualaxtoc	105.0 (16.8)	78.0 (16.2)	80.8 (13.6)
Laguna Negra	114.2 (22.9)	71.6 (18.2)	82.7 (18)
Dwarf mangroves			
Xamach	3.0 (0.4)	8.7 (0.9)	4.9 (0.5)
La Raya	7.1 (0.7)	19.0 (2.2)	10.9 (1.2)
El Playon	5.3 (1.3)	12.2 (3.3)	7.3 (1.9)
Marsh			
Punta Gorda	23.4 (3.0)*	n.a.	11.7 (1.5)
Vigia Chico	18.0 (2.2)**	0.7 (0.3)***	8.5 (1.2)

Data are mean (standard error).

Nine sites were sampled (*n* = 6 plots per site) within coastal wetlands of Sian Ka'an Biosphere Reserve, Mexico. Values are shown as mean (standard error); n.a. = not available.

* aboveground biomass of marsh.

** aboveground biomass of marsh plus mangrove trees.

*** belowground biomass of mangrove trees.

### 3. Downed wood

The mean specific gravity for small wood debris was 0.68±0.03 g cm^−3^. For large wood debris, the mean specific gravity was 0.72±0.03 g cm^−3^ for sound debris, and 0.47±0.03 g cm^−3^ for rotten debris. Considerable amounts of downed wood were only found within the tall and medium mangroves, with a mean of 16.7±4.2 Mg ha^−1^, ranging from 7.0±1.5 Mg ha^−1^ in Laguna Negra to 25.7±4.4 Mg ha^−1^ in Isla Pitaya (Table 4). Mean C mass of downed wood was 8.3±2.1 Mg ha^−1^. Small wood contributed with 36.8% to the downed wood C stock, while large sound wood contributed 27%, and large rotten wood with 36%.

**Table 4:** Biomass (Mg ha^−1^) and C stocks (Mg ha^−1^) of downed wood for tall and medium mangroves.

Site	Small wood	Large wood sound	Large wood rotten	Total down wood	C stock
	(Mg ha^−1^)	(Mg ha^−1^)	(Mg ha^−1^)	(Mg ha^−1^)	(Mg ha^−1^)
Tall mangroves					
Isla Pitaya	9.9 (1.8)	4.8 (2.1)	11.0 (2.7)	25.7 (4.4)	12.9 (2.2)
Cayo Culebra	5.6 (1.2)	7.1 (1.9)	8.7 (2.1)	21.4 (2.8)	10.7 (1.4)
Medium mangroves					
Hualaxtoc	4.9 (1.0)	4.2 (2.0)	3.4 (1.3)	12.5 (2.3)	6.3 (1.2)
Laguna Negra	4.2 (0.8)	2.2 (1.1)	0.7 (0.4)	7.0 (1.5)	3.5 (0.7)

Sites were sampled within Sian Ka'an Biosphere Reserve, Mexico. Wood debris was calculated separately for small wood (diameter >2.5 and <7.5 cm), and large sound and large rotten wood (diameter >7.5 cm). Values are shown as mean (standard error).

### 4. Soil

The C content in the soil varied among vegetation types and depth. Soil C was composed of a considerable portion of IC, although its proportion to the total soil carbon stock was variable among vegetation types, sites, and soil depths. Soil C was largely composed of OC in tall mangroves, while soil C in marshes was composed of a mixture of OC and IC, or predominately IC. For example, in the Isla Pitaya tall mangrove OC exceeded 28% at all measured depths, while IC concentrations were <1%. In contrast, OC in Punta Gorda marsh was <4% while IC was >8%. The mean surface (0–15 cm) concentration of OC of all vegetation types was 21.6±3.8% with a range of 3.5% to 32.3% (Table 5). The surface OC content was significantly different among vegetation types, with tall (31.2±0.9%) and medium (28.2±1.7%) mangroves having significantly higher OC than dwarf mangroves (20.2±2.6%) and marshes (5.0±0.6%) (*F*_3, 53_ = 6.39, *p* = 0.037).

**Table 5:** Bulk density, organic (OC) and inorganic carbon (IC) content, and soil carbon stocks, nitrogen (N) and phosphorus (P) concentrations (mg g^−1^), and N and P soil stocks (Mg ha^−1^).

Soil depth (cm)	Bulk density	OC	IC	Soil OC	N	P	N∶P	N mass	P mass
	(g cm^−3^)	(%)	(%)	(Mg ha^−1^)	(mg g^−1^)	(mg g^−1^)		(Mg ha^−1^)	(Mg ha^−1^)
Tall mangroves									
Isla Pitaya									
0–15	0.30 (0.06)	28.8 (2.6)	0.6 (0.1)	139 (26)	15.1 (0.8)	1.35 (0.21)	25 (10)	6.9 (1.4)	0.54 (0.10)
15–30	0.25 (0.03)	29.4 (2.4)	0.5 (0.1)	115 (10)	14.1 (0.4)	0.50 (0.14)	82 (54)	5.3 (0.6)	0.18 (0.08)
30–50	0.26 (0.03)	33.9 (1.7)	0.4 (0.2)	180 (19)	12.9 (0.8)	0.57 (0.41)	125 (110)	6.8 (0.9)	0.28 (0.21)
50–100	0.21 (0.03)	33.5 (1.5)	0.9 (0.1)	368 (48)	12.9 (0.9)	0.23 (0.06)	143 (59)	14.1 (1.9)	0.37 (0.11)
>100	0.23 (0.03)	35.1 (0.8)	0.6 (0.1)	377 (31)	12.0 (1.2)	0.04 (0.00)	610 (82)	14.5 (2.0)	0.04 (0.00)
Total				1166 (94)				47.0 (5.2)	1.39 (0.35)
Cayo Culebra									
0–15	0.15 (0.01)	32.3 (1.0)	1.3 (0.7)	74 (4)	16.6 (0.6)	0.49 (0.05)	76 (18)	3.8 (0.2)	0.12 (0.02)
15–30	0.11 (0.01)	27.7 (2.2)	2.8 (1.0)	44 (6)	13.9 (0.9)	0.40 (0.02)	75 (15)	2.2 (0.4)	0.07 (0.02)
30–50	0.22 (0.08)	12.6 (7.8)	5.7 (2.5)	58 (11)	10.8 (2.5)	0.46 (0.09)	50 (12)	2.9 (0.5)	0.13 (0.03)
50–100	0.67 (0.01)	3.2 (0.4)	8.5 (0.2)	333 (38)	3.9 (1.2)	0.22 (0.10)	48 (1)	36.7 (11.8)	0.75 (0.35)
Total				508 (41)				45.7 (11.6)	1.07 (0.30)
Medium mangroves									
Hualaxtoc									
0–15	0.32 (0.08)	27.8 (5.2)	2.4 (1.3)	116 (21)	9.8 (2.0)	0.33 (0.11)	55 (15)	3.9 (0.8)	0.16 (0.01)
15–30	0.41 (0.11)	23.3 (6.4)	3.7 (2.5)	127 (35)	7.5 (2.0)	0.21 (0.05)	59 (50)	3.6 (1.7)	0.09 (0.02)
30–50	0.44 (0.08)	14.8 (6.9)	6.2 (2.4)	125 (27)	4.2 (1.6)	0.21 (0.03)	35 (24)	3.3 (1.6)	0.13 (0.02)
50–100	0.91 (0.11)	1.5 (0.1)	9.4 (0.1)	209 (17)	0.5 (0.2)	0.08 (0.01)	18 (14)	82.2 (26.6)	0.29 (0.04)
Total				577 (71)				93.0 (26.4)	0.67 (0.02)
Laguna Negra									
0–15	0.18 (0.01)	27.6 (6.8)	2.8 (2.0)	81 (3)	13.2 (1.4)	0.33 (0.05)	106 (101)	3.5 (0.3)	0.08 (0.02)
15–30	0.18 (0.17)	21.9 (5.7)	4.3 (1.7)	64 (2)	12.2 (1.7)	0.31 (0.05)	101 (36)	3.1 (0.4)	0.08 (0.01)
30–50	0.30 (0.20)	21.0 (8.3)	3.6 (2.3)	107 (24)	10.3 (2.0)	0.31 (0.01)	84 (9)	3.3 (0.7)	0.08 (0.01)
50–100	0.70 (0.01)	8.1 (3.8)	7.7 (1.6)	244 (19)	2.2 (0.5)	0.07 (0.00)	36 (10)	6.6 (0.9)	0.31 (0.02)
Total				496 (15)				16.4 (1.5)	0.55 (0.03)
Dwarf mangroves									
Laguna Xamach									
0–15	0.40 (0.05)	9.3 (2.2)	5.6 (0.8)	52 (5)	5.4 (0.8)	0.12 (0.01)	94 (18)	2.9 (0.2)	0.08 (0.02)
15–30	0.55 (0.06)	6.0 (1.6)	6.9 (1.2)	48 (4)	2.4 (0.6)	0.10 (0.02)	68 (74)	1.7 (0.2)	0.08 (0.01)
30–50	0.95 (0.02)	2.9 (0.1)	7.9 (0.4)	54 (2)	0.6 (0.1)	0.06 (0.01)	22 (6)	1.1 (0.2)	0.13 (0.02)
50–100	0.74 (0.04)	3.8 (0.5)	8.2 (0.4)	140 (8)	1.1 (0.2)	0.04 (0.01)	59 (16)	4.1 (0.6)	0.16 (0.04)
>100	0.72 (0.04)			113 (20)	1.1 (0.2)	0.03		4.8 (1.3)	0.10
Total				407 (24)				14.5 (1.7)	0.48 (0.05)
La Raya									
0–15	0.18 (0.02)	28.8 (4.6)	1.6 (1.1)	69 (4)	12.4 (1.5)	0.22 (0.10)	134 (34)	3.2 (0.3)	0.06 (0.01)
15–30	0.77 (0.09)	6.0 (1.6)	6.9 (1.2)	67 (8)	1.7 (0.4)	0.06 (0.01)	73 (33)	1.7 (0.3)	0.07 (0.01)
30–50	0.89 (0.03)	3.9 (0.5)	8.1 (0.3)	61 (4)	1.1 (0.1)	0.05 (0.01)	43 (6)	1.9 (0.2)	0.09 (0.01)
>50	0.73 (0.04)	4.8 (1.0)	8.8 (0.8)	121 (42)	1.9 (0.4)	0.03 (0.01)	91 (58)	3.7 (1.0)	0.05 (0.02)
Total				286 (30)				9.1 (0.7)	0.27 (0.01)
El Playon									
0–15	0.16 (0.02)	29.3 (1.2)	0.8 (0.1)	67 (6)	13.5 (1.0)	0.19 (0.05)	162 (79)	3.2 (0.2)	0.05 (0.02)
15–30	0.30 (0.04)	16.7 (6.2)	4.0 (1.7)	69 (3)	6.5 (1.8)	0.08 (0.03)	164 (33)	2.4 (0.3)	0.03 (0.0)
30–50	0.63 (0.02)	4.6 (0.2)	8.6 (0.3)	59 (2)	1.7 (0.4)	0.11 (0.01)	41 (1)	2.1 (0.4)	0.14 (0.02)
50–100	0.39 (0.07)	9.4 (3.2)	4.7 (2.2)	231 (31)	3.3 (1.1)	0.18 (0.03)	48 (20)	6.2 (2.3)	0.30 (0.01)
Total				426 (33)				13.9 (2.3)	0.53 (0.02)
Marsh									
Punta Gorda									
0–15	0.31 (0.10)	3.5 (0.9)	9.4 (0.4)	37 (4)	11.6 (0.5)	0.13 (0.01)	11 (3)	2.4 (0.2)	0.15 (0.01)
15–30	0.34 (0.06)	2.3 (0.4)	8.9 (0.1)	35 (1)	9.7 (0.2)	0.10 (0.01)	8 (1)	2.2 (0.2)	0.17 (0.01)
30–50	0.43 (0.14)	2.3 (0.5)	8.9 (0.1)	46 (5)	7.7 (0.2)	0.09 (0.01)	6 (2)	2.2 (0.2)	0.20 (0.01)
50–100	0.62 (0.04)	1.8 (0.1)	8.9 (0.2)	144 (29)	4.9 (0.1)	0.09 (0.01)	6 (1)	6.0 (1.8)	0.52 (0.08)
Total				238 (41)				12.8 (1.8)	1.03 (0.08)
Vigia Chico									
0–15	0.49 (0.07)	7.2 (1.3)	6.9 (1.3)	48 (7)	9.9 (1.8)	0.59 (0.15)	35 (12)	7.2 (2.5)	0.33 (0.06)
15–30	0.57 (0.14)	7.3 (1.7)	6.1 (1.0)	47 (6)	8.0 (1.2)	0.59 (0.11)	33 (7)	9.6 (5.1)	0.47 (0.16)
Total				95 (10)				16.8 (0.2)	0.80 (0.22)

Nine sites (*n* = 6 plots per site for C and N and *n* = 3 plots per site for P) were sampled within coastal wetlands of Sian Ka'an Biosphere Reserve, Mexico. Values are shown as mean (standard error).

Soil OC was highest in the surface of the soil horizons and decreased with depth (*Z* = −7.63, *p*≤0.0001). In contrast, concentration of IC tended to increase with depth (*Z* = 5.63, *p*≤0.0001)(Table 5). This trend was strongest in dwarf mangroves. For example in the soil of La Raya and El Playon, OC and IC concentration in the surface (0–15 cm) comprised ≈95 and ≈5% of the total C of this depth, respectively. In contrast, at 30–50 cm, the OC contribution was ≈35%, while IC composed ≈65% of the soil C.

Total soil C stocks differed among vegetation types and sites with a rather large range of 95 Mg ha^−1^ at the Vigia Chico marsh to 1,166 Mg ha^−1^ at the Isla Pitaya tall mangrove, where the highest soil C stocks were measured. Intermediate soil C stocks were measured in tall mangroves not associated to freshwater springs (508 Mg ha^−1^) , medium mangroves (496–577 Mg ha^−1^) and dwarf mangroves (286–426 Mg ha^−1^); lowest soil C stocks were measured in marshes (95–238 Mg ha^−1^) (*F*_8, 53_ = 63.57, *p*≤0.0001) (Figure 2B).

### 5. Ecosystem C stocks

The highest ecosystem C stock (1,325 Mg ha^−1^) was measured at Isla Pitaya, a site of tall mangroves associated with a freshwater spring (Table 6). The tall mangroves of Cayo Culebra and the medium mangroves had similar values ranging from ≈580 to 660 Mg ha^−1^. Dwarf mangroves had lower C stocks with values ranging from ≈300 to 430 Mg ha^−1^. The carbon stocks of the marshes were only about 7–18% of that of the tall mangroves of Isla Pitaya, with ecosystem C stocks ≤250 Mg ha^−1^. Overall, there was a significant difference among ecosystem C stocks within vegetation types, with tall, medium and dwarf mangroves having significantly larger C stocks compared to marshes (*F*_3, 53_ = 8.17, *p* = 0.022).

**Table 6:** Ecosystem C stocks (Mg ha^−1^) of nine sites within different vegetation types of coastal wetlands of the Sian Ka'an Biosphere Reserve, Mexico.

Site	C (Mg ha^−1^)
Tall mangroves	
Isla Pitaya	1,325 (134)
Cayo Culebra	648 (41)
Mean	987 (338)
Medium mangroves	
Hualaxtoc	664 (78)
Laguna Negra	582 (33)
Mean	623 (41)
Dwarf mangroves	
Xamach	412 (16)
La Raya	297 (18)
El Playon	433 (30)
Mean	381 (52)
All mangroves	663 (176)
Marsh	
Punta Gorda	250 (30)
Vigia chico	104 (9)
Mean	177 (73)

Values are shown as mean (standard error).

### 6. Salinity and nutrients

Interstitial salinity had a mean of 32.3±8.9‰, with a wide range of values from 5.2‰ at Vigia Chico marsh to 57.2‰ at the dwarf mangroves of Xamach. In general, salinity values were lowest at marshes, and highest at dwarf and medium mangroves (*F*_3, 40_ = 27.42, *p* = 0.011) (Table 1). Low salinity in the sampling area is associated with fresh groundwater flows and springs.

Soil nutrients were variable among sites and vegetation communities. Mean surface N concentration was 10.6±0.7 mg g^−1^. Lowest surface N concentrations (5.4±0.8 mg g^−1^) were measured at Xamach, a dwarf mangrove site with relatively high interstitial salinity dominated by *A. germinans*. Highest N concentrations were measured at Isla Pitaya tall mangroves (15.1±0.8 mg g^−1^) (*F*_5, 52_ = 15.30, *p*≤0.0001). Concentrations of N were not significantly different among vegetation types. Moreover, N concentrations were highest in the first 15 cm, and decreased with depth (*F*_4, 35_ = 8.09, *p* = 0.0003) (Table 5).

Concentrations of surface soil P had a mean of 0.47±0.16 mg g^−1^, with lowest values at Xamach (0.12 mg g^−1^) and highest at Isla Pitaya (1.35 mg g^−1^) (*F*_5, 26_ = 7.90, *p*≤0.0004). Concentrations of P were highest in the first 15 cm and decreased with depth (*F*_4, 104_ = 22.48, *p*≤0.0001). Tall and medium mangroves had higher P concentrations than dwarf mangroves, although the difference was not significant. Marshes had variable concentrations of surface N and P; soil at Punta Gorda marsh was relatively high in N (11.6±0.5 mg g^−1^) and low in P (0.13±0.01 mg g^−1^), while soil at Vigia Chico marsh was relatively low in N (9.9±0.18 mg g^−1^) and high in P (0.59±0.15 mg g^−1^).

Soil N stocks had a mean of 30.1±4.7 Mg ha^−1^ with lowest values found at the dwarf mangroves of La Raya (9.1±0.7 Mg ha^−1^), and highest at the medium mangroves of Hualaxtoc (93.0±26.4 Mg ha^−1^)(*F*_4, 41_ = 10.64, *p*≤0.0001). On the other hand, soil P stocks had a mean of 0.75±0.08 Mg ha^−1^ and were also significantly differently among sites (*F*_3, 20_ = 6.40, *p* = 0.006). There was over a 4-fold difference in the soil P stocks between the dwarf mangrove forest of La Raya (0.27±0.01 Mg ha^−1^) and the tall mangrove forest Isla Pitaya (1.39±0.35 Mg ha^−1^) (Table 5). Soil N∶P ratios were variable across sites, vegetation types and depths (Table 5). The lowest surface N∶P ratios were measured in marshes and highest were measured in dwarf mangroves (*F*_3,27_ = 10.5, *p*<0.001).

**Figure 3:**
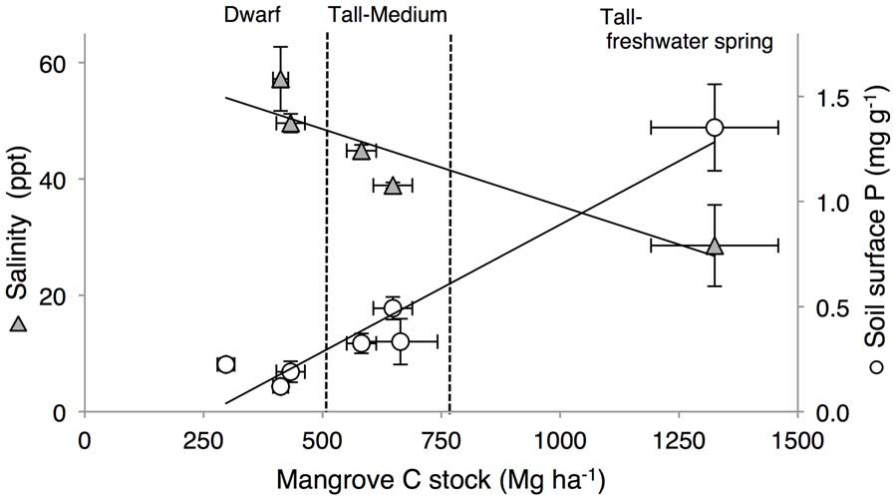
Relationship among mangrove C stocks, interstitial salinity and surface soil phosphorus. Seven mangrove sites were sampled within Sian Ka'an Biosphere Reserve, Mexico; three dwarf, two medium, and two tall mangroves, one of the latter associated to a fresh water spring. Soil phosphorus (P) was measured in the 0–15 cm soil horizon. The correlations are significant with *R* ^2^ = 0.54, *F* = 31.3, *p*<0.0001 and *R* ^2^ = 0.58, *F* = 26.3, *p*<0.001 for C stocks against salinity and soil P, respectively. Collectively, salinity and soil P explained 86% of the variance in mangrove C stocks (*F* = 45.6, *p*<0.001; VIF = 2.2).

Mangrove ecosystem C stocks, as well as C stocks of trees and soil, were closely associated with surface soil P concentrations and were significantly correlated (*R* ^2^ = 0.93, *F* = 62.6, *p* = 0.005; *R* ^2^ = 0.73, *F* = 13.7, *p* = 0.014; *R* ^2^ = 0.58, *F* = 26.3, *p*<0.001, for tree C, soil C, and total C stocks, respectively), such that higher stocks were found in sites with high soil P concentrations. Similarly, C stocks were significantly correlated with salinity and N∶P (*R* ^2^ = 0.54, *F* = 31.34, *p*<0.001; *R* ^2^ = 0.36, *F* = 12.2, *p* = 0.002), such that higher C stocks were found in sites with relatively low salinity and low surface N∶P. We found that the best model to explain C stocks included both soil surface P and salinity (*R* ^2^ = 0.86, *F* = 45.6, *p*<0.001 VIF = 2.2) (Fig. 3). Marshes did not follow this relationship.

### 7. Scaling up

The SKBR has an area of 58,837 ha of mangroves and 112,640 ha of marshes. Overall, 172,176.3 ha of coastal wetlands are found in SKBR (Table 7). These coastal wetlands store 43.2–58.0 million Mg of C, which is the equivalent to 158.6–212.8 million Mg of CO_2e_. Tall mangroves associated with fresh water springs are estimated to comprise 0.4% of the land area and 2.2% of the total carbon stored in the SKBR. Other mangroves comprise 34.2% of the land area and 52–64% of the total carbon stock. Finally, marshes occupy 65.4% of the wetland area and 46% of the carbon stock.

**Table 7:** Area and C stock of coastal wetland vegetation of Sian Ka'an Biosphere Reserve, Mexico.

	Area	Area	C stock	
Vegetation	(ha)	(%)	(Million tonnes)	Vegetation area source
Mangrove forests	58,837	34.2	22.4–37.2	CONABIO (2009)
(dwarf, medium, tall)				
Peten mangroves	700	0.4	0.93	Series III, INEGI (2005)
(tall mangroves associated				
with freshwater springs)				
Marsh	112,640	65.4	19.9	National Forestry
				Inventory, INEGI (2000)
TOTAL	172,176		43.2–58.0	

Mangrove area (dwarf+medium+tall) was obtained from CONABIO [23], “peten” vegetation area (tall mangroves associated with freshwater springs) and marsh area from INEGI maps (2005 and 2000, respectively) [24].

## Discussion

The extensive coastal wetlands of the SKBR comprise a significant C stock. The vegetation community with the highest C stocks per area was a site of tall mangroves associated with a freshwater spring, followed by other tall and medium mangroves and by dwarf mangroves. Marshes had significantly lower C stocks: less than 18% of the stock from the tallest mangrove site. The variability of C stocks within different vegetation types was evident from the aboveground vegetation structure and composition. Mangroves not only have a wide range of ecosystem carbon stocks, but a great variability in structure. The largest proportions of C stocks are belowground, and these require direct measurements. Here we found that the soil C stocks of tall mangroves was 67% larger than that of an average dwarf mangrove, and 85% larger than that of a marsh.

**Figure 4:**
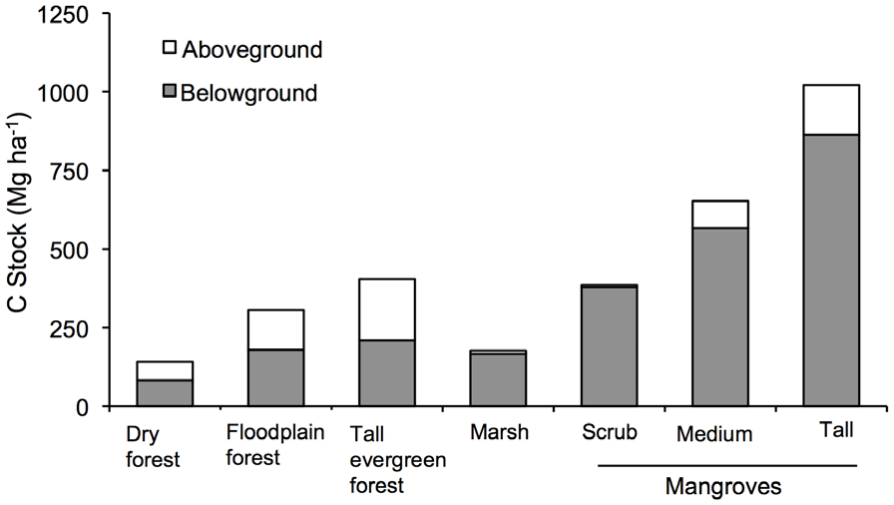
Comparison among C stocks of coastal wetlands of Sian Ka'an Biosphere Reserve with terrestrial forests in Mexico. Terrestrial forests are represented by a dry forest (Chamela, Jalisco [50]), a floodplain forest and an evergreen forests (Los Tuxtlas, Veracruz; [62]). C stocks include aboveground (trees, vines and wood) and belowground (soil and roots) stocks of up to one meter in depth. The tall mangrove forest in the graph was associated to a freshwater spring.

The most widely distributed mangrove ecosystem of the SKBR is the dwarf mangrove. These stands are very unique in structure and are extremely dense (plots as high as 80,000 trees ha^−1^). Others have also reported dwarf mangrove densities of 7,000–20,000 trees ha^−1^ and about 1 m in height [48], [49]. Even more interesting than its unique structure, is the fact that C stocks of the dwarf mangroves were ≈300–430 Mg ha^−1^ (Table 6). Even though the aboveground biomass of the dwarf mangroves is low, the high concentration of C in the surface soil horizons resulted in a relatively large ecosystem C stock. These stocks exceed that of a Mexican tropical dry forest with trees of up to 15 m in height (118–135 Mg ha^−1^; [50]) (Fig. 4).

Highest C stocks of the sites sampled in this study were measured at Isla Pitaya. The Isla Pitaya mangroves were within a zone of the Reserve that is influenced by groundwater discharges developed both by springs feeding local sinkholes and waterways sourced inland [25], [51]. As a result, the site is characterized by low salinity and relatively high soil nutrients. This unique hydrology favors not only the development of mangrove trees but of a distinctive vegetation assemblage, locally known as “Petenes”. This vegetation type was rare in the Reserve (<0.5% of the area), however it contained the highest C stocks per hectare. Carbon stocks of other mangroves were also significant (mean of 506 Mg ha^−1^) and exceeded those of a tropical tall evergreen forest in Mexico (403 Mg ha^−1^ in Los Tuxtlas, Veracruz [52]) (Fig. 4). Tall mangroves in SKBR are comparable, while medium and dwarf mangroves are at the lower end, of the reported range by Donato et al. [1] for mangroves of the Indo Pacific Region (1,023 Mg ha^−1^). As far as we know, this is the first report of ecosystem C stocks of coastal wetlands for the American tropics. Overall, our results support the conclusion of Donato et al. [1] that the unique environment of mangrove forests, including those measuring less than 1 m in height, contain exceptionally high C stocks.

The vegetation composition and structure of coastal wetlands is determined by a suite of environmental parameters, including topography, frequency of inundation, salinity, nutrient concentration, sediment type, and inter species competition [53], [54]. At SKBR we found that high soil P, low N∶P and low salinity were associated with higher C stocks in mangroves. Highest C stocks were measured in a site where mangroves were associated with a freshwater spring (Isla Pitaya) and lowest C stocks were measured in saline (>50‰) dwarf mangroves. The former site was dominated by *L. racemosa*, a species that usually grows in low salinity soils. The close associate of C stocks with soil P was expected, as the productivity of this karstic region is strongly P limited [16], [55]. Coastal wetlands in the Yucatan Peninsula that are relatively enriched with P have the highest litterfall production rates [17], and likely, the highest accumulation of OC in the soil [56], and thus, the highest C stocks. This study suggests that in karstic regions, P limits C sequestration and accretion potential.

There was no clear relation of the C stocks of sampled marsh communities to either salinity or soil nutrients. Both sampled marsh sites had relatively low C stocks, low interstitial salinity (<10‰) and variable nutrient concentrations (Punta Gorda was relatively enriched with N and Vigia Chico with P). Both marshes had lower surface N∶P compared to mangroves, which is a common trait of freshwater wetlands and could indicate N limitation [57]. The marsh at Punta Gorda was dominated by *T. domingensis* and the marsh at Vigia Chico by *C. jamaicense*. The former usually grows on nutrient rich-areas with long flooding periods, while the latter dominates in areas with low nutrient concentrations and occasional drying [28], [58]. The soil C stock at Vigia Chico was >50% lower than that of the Punta Gorda site, which could be a result of C losses during the dry season due to a decrease in C uptake during this period [59], increases in aerobic mineralization [60] and fires (natural and human caused [61]). We make these tentative interpretations of marsh C stocks based upon data of only two sampling sites; obviously more data are needed. However, it is possible that C stocks in marshes are more strongly affected by inundation regimes than by salinity or nutrient availability.

The soil N stocks from our sampling sites ranged from 9–93 Mg ha^−1^, which are higher compared to those from a tall evergreen forests in Mexico (16–20 Mg ha^−1^ [62]) and much higher than those measured in the tropical wet forest of the Amazon basin (0.2–2.3 Mg ha^−1^ [63]). On the other hand, soil P stocks of SKBR ranged between 0.3–1.4 Mg ha^−1^, values that are higher than P stocks (measured as total active P) in tropical mountain forests in Borneo (0.04–0.41 Mg ha^−1^ [64]). It is possible that nutrient stocks, such as C stocks, are significantly larger in mangroves compared to other tropical ecosystems.

There are some limitations to our C stock estimations, such as complications when sampling deep soil horizons and up scaling to large areas. While most of our sites had organic soils <1 m, one of our sites - Cayo Culebra- had an organic soil horizon that exceeded >2 m in depth. Deep organic soils are difficult to sample, and may result in underestimations of the total C stocks in some mangroves. The second complication is probably more important, the up-scaling of site-specific C stock estimations to large areas. Detailed maps of mangroves are not available for many regions, and although the map available for Mexico [23] is considered accurate, we had difficulty distinguishing between vegetation types among mangroves (tall vs. medium vs. scrub mangroves). Furthermore, it would be desirable to distinguish between marshes dominated by *T. domingensis* and those by *C. jamaicense* as the former may have almost twice as much C than the latter (250 vs 104 Mg ha^−1^). These uncertainties resulted in estimations with an error of about 31% for the Reserve, despite the fact that C stock measurements within plots and across vegetation types were highly replicable. In this study we provide the C stock of the SKBR within a range of 43.2–58.0 millions of Mg of C. Future detailed maps of vegetation types could narrow the range of the estimation.

The C stocks in SKBR, store the equivalent of about 185.7 million Mg CO_2e_, which is almost half (40–46%) of the C emissions of Mexico during 2009 (399.7 million Mg of CO_2e_, [65]). This means, that if destroyed by some combination of land use and climate change effects, the coastal wetlands of SKBR alone would release about half of the annual emissions of the whole country as CO_2e_. This would come from a site that only comprises 0.09% of the land area of the country. The coastal wetlands at SKBR are currently protected, but they are still affected by sea level rise, changes in tropical storm intensity, road construction, freshwater extraction and pollution of coastal waters due to increased tourism [15], [66]. As a result, there are varying degrees of alterations in hydrology, increases in salinity and nutrient availability. Increases in these disturbances and stresses could modify the integrity of vegetation communities including the function of C storage within the Reserve. Rates of C sequestration and hence C stocks might temporarily increase as a result of higher production with increased P availability [17] or through landward migration of mangroves into marsh areas [67]. However, C stocks might decrease as a result of flooding, storm surges or due to saltwater intrusions into freshwater springs. Effective climate change mitigation and adaptation strategies should aim at maintaining and restoring the exceptionally large C stocks as well as other ecosystem services provided by coastal wetlands in this Reserve and across the Yucatan region.
